# Using Self-regulation to Successfully Overcome the Negotiation Disadvantage of Low Power

**DOI:** 10.3389/fpsyg.2017.00271

**Published:** 2017-03-14

**Authors:** Andreas Jäger, David D. Loschelder, Malte Friese

**Affiliations:** ^1^Department of Psychology, Saarland UniversitySaarbrücken, Germany; ^2^Institute of Strategic Human Resource Management, Leuphana University of LüneburgLüneburg, Germany

**Keywords:** negotiation, power, self-regulation, if-then plans, setting goals

## Abstract

A plethora of studies has demonstrated that low-power negotiators attain lower outcomes compared to high-power negotiators. We argue that this low-power disadvantage can be conceptualized as impaired goal attainment and that self-regulation can help to overcome it. Three experiments tested this assertion. In Study 1, low-power negotiators attained lower profits compared to their high-power opponents in a face-to-face negotiation. Negotiators who set themselves goals and those who additionally formed if-then plans prior to the negotiation overcame the low-power disadvantage. Studies 2 and 3 replicated these effects in computer-mediated negotiations: Low-power negotiators conceded more than high-power negotiators. Again, setting goals and forming additional if-then plans helped to counter the power disadvantage. Process analyses revealed that negotiators’ concession-making at the start of the negotiation mediated both the low-power disadvantage and the beneficial effects of self-regulation. The present findings show how the low-power disadvantage unfolds in negotiations and how self-regulatory techniques can help to overcome it.

## Introduction

Imagine a job applicant who is about to negotiate her salary with a prospective employer. Her obvious goal is to negotiate a good salary. She knows that there are dozens of other applicants the employer could hire instead of her, leaving her in a low-power position. When the negotiations begin, the employer opens with a low-ball offer that would pay the applicant much less than necessary for a decent living. This highly prevalent real-life example illustrates a key question: How can negotiators craft a profitable deal despite a low-power position? Abundant research suggests that low power in negotiations results in unfavorable outcomes. Since crafting profitable deals is a key goal for negotiators, in the present research, we propose that this low-power disadvantage can be understood as impaired goal attainment. We argue that self-regulation techniques such as setting goals and forming if-then plans, which are geared toward improving goal attainment, can help low-power negotiators to improve their outcomes and, hence, overcome the perils of low power. From a theoretical perspective, the present studies extend the literature by showing when and how the low-power disadvantage unfolds and how self-regulatory techniques can help to overcome it.

## Low Power in Negotiations

Power is one of the most fundamental factors to shape the course and outcome of negotiations (Fisher et al., 1991; Thompson et al., 2010; Overbeck and Kim, 2013). From union-management negotiations, to salary negotiations, to parents discussing bedtime with their children, it is difficult to imagine a negotiation in which power does not play a vital role. Previous research defined low power as a structural state of having a comparatively limited control over scarce resources and therefore being dependent on another party (Thibaut and Kelley, 1959; Emerson, 1962; Fiske, 1993; Magee and Galinsky, 2008). Our common sense tells us that high-power parties will prevail in negotiations with low-power parties. A host of experimental studies confirms this notion, showing that low-power negotiators reach poorer negotiation outcomes: They claim less value (De Dreu, 1995), are exposed to more attempts of intimidation (De Dreu, 1995), are less willing to initiate negotiations (Volkema et al., 2013), are less likely to make first offers (which generally yields a bargaining advantage; Magee et al., 2007), concede more (Van Kleef et al., 2006), and – most importantly – end up with less profitable deals compared to their more powerful opponents (Dwyer and Walker, 1981; Pinkley et al., 1994; Brett et al., 1996; Giebels et al., 2000; Kim and Fragale, 2005; Kim et al., 2005; Wolfe and McGinn, 2005).

Surprisingly, whereas abundant research has described how low power impairs negotiation outcomes, little research has addressed how parties can overcome this low-power disadvantage. Doing so would not only benefit low-power negotiators but could also shed light on the underlying processes that cause the low-power disadvantage in the first place. In other words, a means to overcome the low-power disadvantage offers a theoretical contribution as it may tackle a meaningful mediator that underlies the low-power disadvantage. The present article aims to identify such a mediator.

Following from the reality that crafting profitable deals is one of the major goals in negotiations (Allred, 2000), it is rather straightforward to conceptualize the low-power disadvantage (impaired outcome either relative to an opponent or to the optimal outcome) in negotiations as an instance of impaired goal attainment. Hence, crafting a suboptimal deal represents impaired goal attainment. Self-regulation research offers a variety of tools that improve goal attainment, a number of which seem feasible for negotiations (e.g., setting goals, if-then plans, mental contrasting; Friese et al., 2011; Jäger et al., 2015). Applying self-regulation tools might thus be a promising approach to overcome the low-power disadvantage. Corroborating the notion that improving self-regulation might help with the low-power disadvantage, some prior evidence already suggests that low-power negotiators set less ambitious goals than high-power negotiators (Wolfe and McGinn, 2005), and fail to initiate goal-congruent behavior (Galinsky et al., 2003; Guinote, 2007; Steidle et al., 2013). Both of these behaviors reflect deficient self-regulation.

## Self-Regulation in Negotiations

Self-regulation research investigates how people pursue goals and which factors promote or impede goal attainment (Mann et al., 2013; Vohs and Baumeister, 2016). Self-regulation comprises all efforts to alter one’s thoughts, emotions, and behavior in order to improve goal attainment, including, for example, setting goals, planning, implementing plans, and shielding plans from competing processes (Mischel et al., 1996; Fujita, 2011).

One particularly effective self-regulation technique is to set goals (Inzlicht et al., 2014; Locke and Latham, 2015). Goals are the deliberate formulation of what an individual wants to attain (Locke et al., 1981), guiding all subsequent efforts, encouraging persistence, facilitating strategy development, and mobilizing on-task effort (Locke et al., 1981). A meta-analysis has shown that – independent of power differences between negotiators – setting goals is overall a successful strategy to improve negotiation outcomes (Zetik and Stuhlmacher, 2002). Together with the finding that low power leads people to form relatively unambitious goals (Wolfe and McGinn, 2005) this leads to the prediction that setting goals should help low-power parties to overcome the low-power disadvantage and attain more profitable outcomes. Low-power negotiators not only set unambitious goals, but also have difficulties initiating goal-congruent actions (i.e., goal pursuit; Steidle et al., 2013). Those findings might be rooted in low-power individuals’ general difficulties to initiate actions (compared to high-power individuals; Galinsky et al., 2003). Moreover, low power increases situational pressure, leading low-power individuals to act in accordance with situational cues (Galinsky et al., 2008). This may prevent low-power negotiators from initiating goal-directed actions. The self-regulation technique of forming if-then plans (Gollwitzer, 1999) may be an effective means to address this concern. If-then plans specify when and how to carry out goal-directed behavior, according to the pattern, “If situation X arises, then I will do Y” (Gollwitzer, 1999). In doing so, if then-plans extend pre-set goals (“My goal is to obtain Z”) and considerably improve goal attainment beyond setting goals alone (see Gollwitzer and Sheeran, 2006, for a meta-analysis). They help initiating goal-directed actions even against strong situational pressure and habits (Armitage, 2004, 2007; Holland et al., 2006).

If-then plans work by creating a mental link between the critical situation (the *if*-part) and the desirable behavior (the *then*-part; Webb and Sheeran, 2006, 2008; Achtziger et al., 2012). They increase the perceptual readiness to recognize critical situations and to carry out the a-priori defined behavior effortlessly (Webb and Sheeran, 2006, 2008; Achtziger et al., 2012). If-then plans are capable of implementing complex mental processes (Henderson et al., 2007; Wieber et al., 2015). A key advantage of this technique is that the *If-X-then-Y* structure is very flexible and can be easily adapted to a variety of settings, rendering these plans a feasible tool in the dynamic context of negotiations (Trötschel and Gollwitzer, 2007; Kirk et al., 2011; Wieber et al., 2014).

The above considerations lead to the question which goal-directed behavior low-power negotiators should implement to overcome their disadvantage. As mentioned above, low power leads to poor outcomes (e.g., a seller agrees to a low selling price). Based on research that identifies the back and forth of offers and counteroffers as the most basic building block of negotiations (e.g., Pruitt and Syna, 1985; Weingart et al., 1990; Prietula and Weingart, 2011) and a host of studies that speak to the importance of refraining from large early concessions (e.g., Galinsky and Mussweiler, 2001; Loschelder et al., 2014a,b, 2016), it can be said that a poor outcome implies at least one of three shortcomings in dyadic negotiation behavior: (1) parties fail to make self-serving offers, (2) parties fail to reject unfavorable (counter) offers, or (3) parties make too large and potentially premature concessions. All three shortcomings could be addressed via setting goals (e.g., *“I will negotiate tenaciously”*) and be further refined with additional if-then plans (e.g., *“If my opponent makes a bad offer, then I will reject it”*). We will identify in the subsequent studies which of these behaviors accounts for the power disadvantage.

## Overview

The present research examines whether and how the negotiation disadvantage of low power can be overcome by self-regulation techniques. In Study 1, low-power negotiators were equipped with goals or additional plans to face a high-power opponent in a face-to-face negotiation. In Studies 2 and 3, low-power negotiators were equipped with goals or additional plans to face a high-powered opponent in a computer-mediated negotiation. Arguably, face-to-face negotiations constitute the default case for real-world negotiations in many contexts and domains. Computer-mediated negotiations became increasingly more important in the last decade (e.g., ebay.com and alibaba.com had a combined net revenue of over $20 billion in 2014) ^1^. Both forms of negotiations are by far more prevalent than any alternative form of negotiation (e.g., written exchange in offline contexts).

Face-to-face and computer-mediated negotiations differ in a number of factors. Most importantly, computer-mediated negotiations allow for only a reduced bandwidth of communicational cues to be sent and received as compared to face-to-face negotiations (Stuhlmacher and Citera, 2005). To illustrate, whether negotiators face each other or communicate via a computer has distinct impacts on negotiation behavior (e.g., rapport building; Arunachalam and Dilla, 1995), parties’ interpersonal perceptions (e.g., judgment accuracy; Drolet and Morris, 2000), and on negotiation outcomes (Croson, 1999; Morris et al., 2002). Accordingly, conceptually replicating results in both contexts should increase confidence in the proposed effects and their ecological validity. In addition to generally investigating the disadvantage of low power and the advantage of self-regulation, we examine how the low-power disadvantage emerges and, in a second step, how self-regulation techniques help to overcome this low-power disadvantage. Specifically, we contrast two mechanisms potentially mediating the effect of power and self-regulation techniques in Studies 2 and 3. We examined (a) if power affects negotiation outcomes, and (b) if the two self-regulatory antidotes counter this effect throughout the whole negotiation or mainly at the very beginning of negotiations. Therefore, we contrasted parties’ first concessions and concessions in the subsequent negotiation process.

## Study 1: Overcoming Low Power in a Face-To-Face Negotiation

Study 1 examined the hypothesis that low-power negotiators will craft more profitable deals by setting goals and forming if-then plans. In a *control* condition a high-power and a low-power negotiator negotiated without either party receiving self-regulatory help. In two further experimental conditions low-power negotiators either specified a goal (*goal* condition) or an additional if-then plan (*plan* condition). We predicted that in the *control* condition high-power negotiators would achieve more beneficial negotiation outcomes than their low-power counterparts (Hypothesis 1). The outcome difference between high- and low-power parties should be reduced in the *goal* condition (Hypothesis 2a) and even further reduced in the *plan* condition (Hypothesis 2b). Note that not fully crossing power and self-regulation allows focusing on the main research question – does self-regulation help to overcome the detriments brought about by low power in negotiations? We wish to clarify that this constrained design does not allow for an investigation of the interaction of power and self-regulation. This latter question is an interesting topic in its own regard, but it was not crucial for present purposes. The same holds true for Studies 2 and 3.

### Method

#### Ethics Statement for All Studies

At our institution, the studies reported here are considered minimal risk studies that did not require formal ethical approval. Researchers are given responsibility to conduct their research in line with ethical guidelines provided by the national psychological association that all researches are required to follow. Participants were not fully informed about the goals of the respective study beforehand, because this would have undermined the effects of the experimental manipulations. All participants were thoroughly debriefed after taking part in a study.

#### Participants and Design

One hundred and thirty-eight participants were recruited on the campus of Saarland University in exchange for €5. Additionally, participants took part in a lottery of a €25 gift certificate for a popular internet store that was raffled among the five participants with the highest individual gains at the end of the negotiation. Participants were randomly assigned to the (1) control, (2) goal, or (3) plan condition. Three dyads (six participants) were excluded from the analyses because one of the participants did not make a plan or did not set a goal (*n* = 2) or because one of the participants reported having detailed *a priori* knowledge about the study (*n* = 1). This left a final sample of 66 dyads (132 participants, *M*_age_ = 23.79, *SD* = 3.82).

#### Procedure

In each session, two participants engaged in a sales negotiation. Negotiators were seated opposite to each other at a rectangular bargaining table. Buyer and seller roles were assigned randomly to their respective role. After welcoming participants, the experimenter gave a summary of the negotiation task and reminded participants that negotiating successfully would increase their chances of winning the €25 gift certificate. Participants had 9 min to read their instructions and to familiarize themselves with their payoff table (**Table 1**). Participants then negotiated for up to 9 min. Pretests had shown this period of time to be sufficient for finding an agreement on all items. At the end of the negotiation, participants noted their agreement or an impasse on a contract sheet, filled out a post-experimental questionnaire, were debriefed, thanked, and paid.

**Table 1:** Payoff table for the buyer (high-power negotiator) and seller (low-power) in Study 1.

Negotiation issues											
**Price**			**Delivery time**			**Warranty period**			**Maintenance contract**		
**Agreement Level (Million)**	**Points Buyer**	**Points Seller**	**Agreement Level (months)**	**Points Buyer**	**Points Seller**	**Agreement Level (years)**	**Points Buyer**	**Points Seller**	**Agreement Level (years)**	**Points Buyer**	**Points Seller**
1.4	24	–24	0	6	6	0	–36	18	0	18	–36
1.5	20	–20	1	5	5	1	–30	15	1	15	–30
1.6	16	–16	2	4	4	2	–24	12	2	12	–24
1.7	12	–12	3	3	3	3	–18	9	3	9	–18
1.8	8	–8	4	2	2	4	–12	6	4	6	–12
1.9	4	–4	5	1	1	5	–6	3	5	3	–6
2.0	0	0	6	0	0	6	0	0	6	0	0
2.1	–4	4	7	–1	–1	7	6	–3	7	–3	6
2.2	–8	8	8	–2	–2	8	12	–6	8	–6	12
2.3	–12	12	9	–3	–3	9	18	–9	9	–9	18
2.4	–16	16	10	–4	–4	10	24	–12	10	–12	24
2.5	–20	20	11	–5	–5	11	30	–15	11	–15	30
2.6	–24	24	12	–6	–6	12	36	–18	12	–18	36

#### Negotiation Task

Each dyad negotiated the sale of ten robots in an industrial context (Siegel and Fouraker, 1960; Pruitt and Lewis, 1975). Buyers were placed in a high-power position, whereas sellers were placed in a low-power position (see below). Both parties learned that they should negotiate the best possible deal for their company (scoring as many points as possible). The negotiation revolved around four issues (i.e., price, warranty period, maintenance contract, delivery time; see **Table 1**). Each issue had 13 potential levels of agreement, with points reflecting the value of each agreement level. The maximum of points per participant was +86; the minimum was -86 (**Table 1**).

Participants were told that they had to come to an agreement on all four issues. In case of a non-agreement on at least one issue the negotiation would be considered an impasse and both parties would receive the points described as their alternative to negotiating an agreement (see below).

#### Experimental Manipulations

To realize a particularly strong power manipulation – which self-regulation techniques were tested to overcome – we combined three established power manipulations. First, high-power negotiators (buyers) were asked to write about a situation in their lives in which they had power over another person (Galinsky et al., 2003). The low-power negotiators did not write about such an incident but were given time to take notes to prepare for the negotiation. Second, the buyer role was described as more powerful than the seller role (Pinkley et al., 1994): Buyers read that they were in a superior position (i.e., buyers’ market; see Trötschel et al., 2015). Sellers read that they were in an inferior position. Third, negotiators’ alternatives to crafting an agreement were manipulated in that high-power buyers had a good alternative: They could buy the product from another company (equivalent to +10 points as final outcome; Pinkley et al., 1994). By contrast, sellers had no alternative to a negotiated agreement (no alternative buyers, equivalent to 0 points as final outcome).

To manipulate self-regulation, low-power negotiators in the *goal* condition read that past research has shown people to benefit from setting goals. Parties were asked to set themselves the following goal: “I will achieve as many points as possible.” In the *plan* condition, low-power parties additionally read that a specific plan on how to implement goals was beneficial. They were asked to form the following plan: “If my opponent makes an unfavorable offer, then I will reject it and make a mutually beneficial counteroffer.” Low-power negotiators in the *control* condition received no self-regulatory help.

#### Dependent Variables

To quantify the extent of the low-power disadvantage, the difference in points for low-power and high-power parties served as the main dependent variable. Negative scores indicate that high-power parties claimed more points than low-power opponents; positive scores indicate that the low-power negotiator earned more points than the high-power opponent.

After the negotiation ended, participants rated their feelings of power during the negotiation with four items in a post-negotiation questionnaire (e.g., “I felt powerful in the course of the negotiation,” “Compared to my opponent I felt superior,” ranging from 1 = *strongly disagree* to 7 = *strongly agree*, α = 0.79) as a manipulation check. Finally, demographic data were obtained (e.g., age, sex, field of study).

### Results

Throughout this manuscript, outliers more than 2.5 *SD*s from the respective condition mean of the main dependent variable were excluded.

#### Manipulation Check

A paired *t*-test between the high-power (*M* = 4.92, *SD* = 0.87) and the low-power negotiator (*M* = 3.86, *SD* = 0.91) within a dyad yielded a significant difference in self-reported power in the *control* condition (*t*[20] = 3.65, *p* = 0.002, *d* = 1.64). This power difference was not found for the two self-regulation conditions (both *t*s < 1.24, both *p*s > 0.230). The overall ANOVA showed a significant Power × Self-regulation interaction only, *F*(2,60) = 4.54, *p* = 0.015, $\eta_{p}^{2}$ = 0.13 [power main effect: *F*(1,60) = 1.07, *p* = 0.304, $\eta_{p}^{2}$ = 0.02]. These findings indicate that, first, the power manipulation was effective in the control condition. Second, participants in the *goal* and *plan* conditions, in retrospect, did not feel less powerful than their opponents – possibly because the success of the self-regulation techniques led these participants to feel equally powerful as their more powerful opponents. We will return to this finding in the discussion of Study 1.

#### Negotiation Outcomes

**FIGURE 1:**
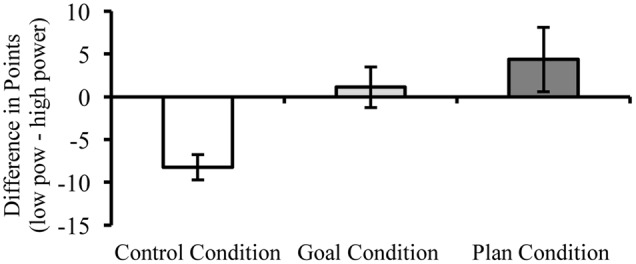
**Mean point difference between the low-power and the high-power negotiator as a function of self-regulatory condition in Study 1.** Error bars indicate ±1 *SEM*.

As expected, high-power negotiators claimed more value than their low-power opponents in the *control* condition, as indicated by a difference score significantly lower than zero (*M*_Δ_ = –8.24, *SD* = 6.80), *t*(20) = -5.55, *p* < 0.001, *d* = -1.21. There was no such effect in the *goal* condition (*M*_Δ_ = 1.15, *SD* = 10.63), *t*(19) = 0.48, *p* = 0.634, *d* = 0.11 and the *plan* condition (*M*_Δ_ = 4.36, *SD* = 17.60), *t*(21) = 1.16, *p* = 0.258, *d* = 0.25. The ANOVA revealed a significant main effect for the experimental condition, *F*(2,60) = 5.71, *p* = 0.005, $\eta_{p}^{2}$ = 0.16. Further analyses showed that low-power parties in both the *goal* (*t*[32.05] = 3.35, *p* = 0.002, *d* = 1.18) and the *plan* (*t*[27.37] = 3.12, *p* = 0.004, *d* = 1.19) condition outperformed their low-power equivalents in the *control* condition. In line with Hypotheses 2a and 2b, low-power negotiators reached increasingly beneficial outcomes from the *control* condition, to the *goal* condition, to the *plan* condition. The linear trend was highly significant, *F*(1,60) = 10.69, *p* = 0.002 (**Figure 1**). Despite this linear trend, the *plan* condition was only descriptively, but not significantly, more successful than the *goal* condition, *t*(35.01) = 0.72, *p* = 0.474, *d* = 0.24.

### Discussion

In line with prior research, low-power negotiators achieved lower profits than their high-power opponents (Pinkley et al., 1994). Setting a goal or setting a goal and additionally forming a plan on how to implement goal-consistent action eliminated the power disadvantage (**Figure 1**). These findings support the assumption that the self-regulation techniques of setting goals and forming if-then plans can help to overcome the low-power disadvantage.

The manipulation check revealed that low-power negotiators in the two self-regulation conditions did not report significantly lower feelings of power than their more powerful opponents. This surprising finding may have resulted from the fact that the manipulation check was assessed *after* a deal had been made, possibly causing beneficial deals to mitigate the subjective power disadvantage. In line with this assumption, regression analyses showed that outcome differences between high-power and low-power negotiators were a significant positive predictor for self-reported feelings of power reported *after* the negotiation (β = 0.02, *t*[61] = 2.02, *p* = 0.048). The more beneficial the outcome for the low-power negotiator, the more powerful they felt. Together with the substantial differences in reported power in the control condition this suggests that the power manipulation may initially have been effective in all conditions, but that low-power negotiators in the self-regulation conditions learned during the course of the negotiation that things were going well for them (i.e., that they were not powerless after all), which in turn increased subjective feelings of power.

## Study 2: Overcoming Low Power in Computer-Mediated Negotiations

The purpose of Study 2 was threefold. First, we sought to generalize the effects obtained in Study 1 to the context of computer-mediated negotiations. Many negotiations occur online – via e-mail and instant messaging (Katsh and Rifkin, 2001; Dorado et al., 2002; Tyler, 2004), or over trading and auction websites (e.g., ebay, craigslist, or alibaba). We relied on one of the most widely used computer-mediated negotiation paradigms (De Dreu and Van Lange, 1995; Sinaceur and Tiedens, 2006; Van Kleef and Côté, 2007; Steinel et al., 2008; Van Kleef and De Dreu, 2010). Previous research has shown that in this paradigm low power constitutes a negotiation disadvantage that leads parties to make lower final offers (larger concessions; Van Kleef et al., 2006). We expected that low-power negotiators would make lower final offers than high-power negotiators (Hypothesis 1) and that this low-power disadvantage would be reduced in the *low power+goal* (Hypothesis 2a) and in the *low power+plan* condition (Hypothesis 2b).

Second, Study 2’s design addresses a possible confound in Study 1. In Study 2, we assigned both high- and low-power negotiators to the same role (instead of buyer versus seller as in Study 1) to rule out that the effects from Study 1 were actually role effects rather than power effects.

Third, we sought to cast light on how and – more importantly – when power unfolds its effect in the negotiation process. This key question has been largely neglected up to this point. At least two different mechanisms are conceivable. On one hand, power may affect the very beginning of a negotiation in that low-power negotiators may make particularly large concessions early on during the negotiations – a disadvantage that may be difficult to overcome during the remaining negotiation. On the other hand, the difference between high power and low power could take time to unfold. Many negotiation experts advise to first build rapport at the beginning of a negotiation (Moore et al., 1999; Nadler, 2004; Fisher and Shapiro, 2005). Following such advice might lead negotiators, even powerful ones, to start moderately. Hence, high versus low-power negotiators might not differ in their initial concessions but rather in their concession-making during the negotiation process. Identifying the leverage point of power differences is not only of theoretical importance but could also prove helpful with respect to assisting low-power negotiators to overcome their disadvantage. Hence, we also explored the extent to which self-regulation affected parties’ initial and subsequent concession-making during the negotiation.

### Method

#### Participants and Design

Ninety-five participants of {Saarland University} (*M*_age_ = 23.45, *SD* = 5.99) were randomly assigned to a (1) *high power*, (2) *low power*, (3) *low power+goal*, or (4) *low power+plan* condition.

#### Procedure

In each session, up to four participants were seated in separate cubicles equipped with desktop computers. First, posture was manipulated (see below; Huang et al., 2011). All subsequent instructions were presented on the computer screen. Participants were informed that the study was conducted together with the university’s management department and that the opponent was located in the management building during the negotiation. In reality, participants negotiated with a computer program that standardized and simulated the counterpart’s behavior (Van Kleef and Côté, 2007). Next, the self-regulation manipulations were established (see below).

At the start of the negotiation, participants saw a “Waiting for server” message while the program allegedly connected participants from different departments. Negotiations ended after six rounds of offers and counteroffers. After it had ended, it was explained to the participants that the study’s goal was to examine early phases of negotiations to ensure that participants were not irritated due to the abrupt ending. Then, participants completed a post-experimental questionnaire before being probed for suspicion. They were thoroughly debriefed, paid, and thanked. The experimenter apologized for the deception.

#### Negotiation Task

Participants assumed the role of a job candidate negotiating with a prospective employer. The job negotiation featured three issues: Salary, vacation days, and working hours (modeled after Van Kleef et al., 2004; Sinaceur et al., 2011). Each of the three issues had nine agreement levels yielding between one and nine points (**Table 2**). The maximum of points to be earned was 27, the minimum was 3. Participants were given a payoff chart and each negotiation offer and counteroffer was converted into a point sum on participants’ computer screen.

**Table 2:** Payoff table for participants (employees) in Studies 2 and 3.

	Negotiation issues					
Points	Wage (€)		Vacation days		Working hours	
	Buyer	Seller	Buyer	Seller	Buyer	Seller
1	2400	3200	25	33	43	35
2	2500	3100	26	32	42	36
3	2600	3000	27	31	41	37
4	2700	2900	28	30	40	38
5	2800	2800	29	29	39	39
6	2900	2700	30	28	38	40
7	3000	2600	31	26	37	41
8	3100	2500	32	27	36	42
9	3200	2400	33	25	35	43

Participants were instructed to earn as many points as possible and learned that their points would be converted into lottery tickets—with their chances of winning a €25 gift certificate from a popular internet store being directly proportionate to their negotiated points. Only dyads with an agreement were to participate in the lottery. Thus, the task resembled the mixed-motive nature of most real-life negotiations: Parties strove to maximize their outcomes while also needing to cooperate to reach an agreement (Sinaceur et al., 2011).

Negotiators proposed offers and counteroffers via a computer interface by moving sliders for each negotiation issue. They had the opportunity to communicate via short text messages. The employer (the computer-simulated opponent) made the first offer. Thereafter, job candidate and employer made alternating offers and counteroffers. The negotiation ended (1) when an offer from the participant equaled or exceeded the next pre-programmed offer, (2) when the participant accepted an offer, or (3) after a maximum of six rounds (De Dreu and Van Lange, 1995; Van Kleef and Côté, 2007). The simulated employer made the following offers for salary, vacation days, and working hours, respectively (in points for the participant): 2–3–2 (Round 1), 2–3–3 (Round 2), 2–4–3 (Round 3), 3–4–3 (Round 4), 3–4–4 (Round 5), and 4–4–4 (Round 6). Prior research suggests that this offer pattern leads participants to believe they are negotiating with a real counterpart. It is perceived as intermediate in cooperativeness and competitiveness (De Dreu and Van Lange, 1995).

#### Experimental Manipulations

We again used multiple, established power manipulations to provide a conservative test for the effectiveness of self-regulation techniques. First, a posture manipulation (Carney et al., 2010, 2015; Huang et al., 2011) was realized *prior to* the start of the negotiation. The experimenter informed participants that certain postures would help them concentrate and engage in their role as job candidate. In the *low-power* conditions, participants were asked to vividly imagine being a job candidate without alternatives, while putting their hands under their thighs, placing their legs closely together and looking at the space between their feet for 90 s (Carney et al., 2010). In the *high*-*power* condition, participants were asked to vividly imagine being a job candidate with plenty of alternatives, while placing their feet on the table (legs stretched), crossing their hands behind their head, and looking upward for 90 s (Carney et al., 2010).

Second, we realized a prominent role manipulation of power (Pinkley et al., 1994). High-power negotiators were told that they had numerous job offers, which they could fall back to if this negotiation ended without an agreement. Low-power negotiators were told that the market was very competitive and that they had no alternatives to this particular job. To enhance this second power manipulation, the alleged employer (i.e., the program) sent text messages emphasizing her/his powerful position together with the offers in round 3 and round 5 (De Dreu, 1995). The first expression read: “You will have to make some concessions. There are other well-qualified candidates.” The second expression read: “You should seriously consider whether you want this job or not. I don’t see us reaching an agreement this way.” Participants in the *high-power* condition did not receive these messages. To increase experimental realism, the computer program sent the following messages in all four conditions after rounds 1 and 2, respectively: “Here is my first offer” and “Hmm. I will offer this:” No messages were sent after rounds 4 and 6.

To manipulate self-regulation, participants in the *low power+goal* condition read prior to the negotiation that past research has shown people to benefit from being tenacious in negotiations. They were asked to set themselves the following goal: “I will negotiate tenaciously and claim as many points as possible.” Participants in the *low power+plan* condition additionally read that a specific plan on how to implement their goal was beneficial. They were asked to form the following plan: “If my opponent makes a request or tries to put me under pressure, then I will not be swayed and budge from my offer in small steps only.”

#### Dependent Variables

Participants’ final offers served as the dependent variable: We examined how much less participants claimed than the theoretical maximum of 27 points.

Follow-up analyses also investigated potential underlying mechanisms of power and self-regulation techniques on final outcomes. As detailed above, the low-power disadvantage could be a consequence of different types of concession-making: Initial concessions or concessions made during the subsequent negotiation process. To disentangle these possibilities, we examined initial concessions (counteroffer in round 1) and subsequent concessions (offer in round 5 minus offer in round 2, controlling for initial concessions).

In a post-experimental questionnaire, four items assessed the effectiveness of the power manipulation (e.g., “I felt powerless in the course of the negotiation”; “Compared to my opponent I felt superior”; 1 = *strongly disagree* to 7 = *strongly agree*, α = 0.80). Additionally, several items asked for demographic data (e.g., sex, age, field of study).

### Results

#### Manipulation Check

As expected, participants in the *high-power* condition felt more powerful than participants in the three *low-power* conditions, *t*(87) = 2.32, *p* = 0.023. The one-way ANOVA was marginally significant, *F*(3,87) = 2.38, *p* = 0.075, $\eta_{p}^{2}$ = 0.08. As expected, the low-power conditions did not differ from each other, *t*s(87) < 1.4, *p*s > 1.90.

#### Final Offers

**FIGURE 2:**
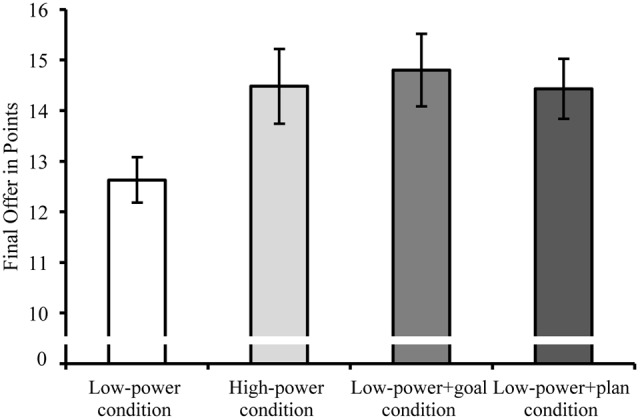
**Study 2: Final offers as a function of power and self-regulatory technique (higher values indicate fewer concessions).** Low-power negotiators made lower final offers than high-power negotiators and low-power negotiators with self-regulatory help. Error bars indicate ±1 *SEM*.

As expected, participants in the *low*-*power* condition (*M* = 12.63, *SD* = 2.22) made less ambitious final offers than participants in the *high-power* condition (*M* = 14.48, *SD* = 3.53), *t*(36.83) = 2.14, *p* = 0.039, *d* = 0.63 (**Figure 2**). Participants in the *low power+goal* condition (*M* = 14.80, *SD* = 3.58) counteracted this low power detriment. They made more ambitious final offers than participants in the *low-power* condition (*t*[40.34] = 2.57, *p* = 0.014, *d* = 0.73) and did not differ from the *high-power* condition, *t*(45.78) = -0.31, *p* = 0.755, *d* = -0.09. Participants in the *low power+plan* condition (*M* = 14.43, *SD* = 2.83) also outperformed the *low-power* condition, *t*(41.78) = 2.43, *p* = 0.019, *d* = 0.71, and did not differ from the *high-power* condition, *t*(41.99) = 0.05, *p* = 0.963, *d* = 0.02, and the *low power+goal* condition, *t*(44.90) = -0.39, *p* = 0.700, *d* = -0.12. The overall ANOVA was marginally significant, *F*(3,91) = 2.45, *p* = 0.069, $\eta_{p}^{2}$ = 0.08.

#### Process Analyses

To shed light on (1) how power differences affect negotiation outcomes and (2) how self-regulation helps to overcome the low-power disadvantage, we examined the competing mediators of initial concessions and subsequent simultaneously (PROCESS macro; Hayes, 2013, Model 4; 5,000 bootstrapped samples). To obtain a pure measure of subsequent concessions and to control for the overlap with initial concessions, we partialled out initial concessions from concessions during the subsequent negotiation. In a first step, we analyzed whether initial or subsequent concessions mediated the detrimental effect of low power versus high power on final outcomes. Condition was coded according to the observed outcome pattern (*low-power* condition = –1, *high-power* condition = +1). There was neither a significant indirect effect through initial, *b* = -0.22, *SE* = 0.27, BC CI_95%_ [-0.758, +0.299], nor through subsequent concessions, *b* = -0.28, *SE* = 0.23, BC CI_95%_ [-0.788, +0.129]. This leaves the question how power affects final outcomes currently unanswered.

**FIGURE 3:**
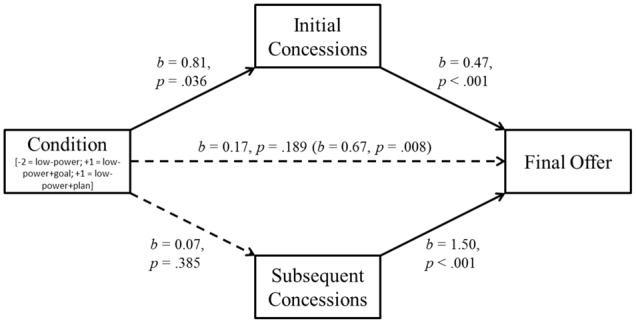
**Study 2: Mediation of the self-regulation effect by initial and subsequent concessions.** The indirect path through initial concessions reaches significance, the path through subsequent concessions does not. Total effect of self-regulation before inclusion of mediators in parentheses.

In a second step, we analyzed whether the beneficial effect of self-regulation was mediated through initial concessions or through subsequent concessions. Again, condition was coded according to the observed outcome pattern (*low-power* condition = –2, *low power+goal* condition = +1, *low power+plan* condition = +1). There was a significant indirect effect through initial concessions on final outcomes, *b* = 0.38, *SE* = 0.19, BC CI_95%_ [+0.023, +0.771]. The indirect path through subsequent concessions was not significant (*b* = 0.11, *SE* = 0.12, BC CI_95%_ [-0.123, +0.358]). These mediation analyses suggest that self-regulatory techniques helped to overcome the low-power disadvantage by means of smaller *initial* concessions (**Figure 3**).

### Discussion

Replicating Study 1 and prior research (Van Kleef et al., 2006), low-power negotiators made more conciliatory final offers than high-power negotiators. Importantly, both setting goals and forming if-then plans counteracted this disadvantage: Low-power negotiators with a goal and those who had additionally formed a plan made more ambitious final offers. Follow-up process analyses investigated the underlying mechanisms for this effect. The analyses suggested that self-regulation helps negotiators to overcome their low-power disadvantage by showing more resistance at the very beginning of a negotiation. One would expect that successfully overcoming the detrimental effect of low power requires low-power negotiators to tackle the same mediator that caused the detrimental effect of low power in the first place. Yet, neither initial nor subsequent concessions mediated the basic effect of power difference. This may have been due to a lack of statistical power and calls for a replication with a larger sample size.

## Study 3

Study 3 sought to conceptually replicate Study 2 in a larger sample. Moreover, the paradigm is geared toward measuring the process of negotiations rather than negotiation outcomes. Final agreements are not measured. As, however, final agreements are an important dependent variable, we assessed negotiators’ absolute limits as a proxy of final agreements.

Furthermore, Study 3 aims to address two possible confounds of Studies 1 and 2. Both in Study 1 and 2, we used several power manipulations to ensure a particularly strong manipulation of power. Those manipulations might have interacted in an unforeseen manner, manipulating more than power. Accordingly, we used a single power manipulation in Study 3. Moreover, low-power participants in Study 2 received more text messages than high-power negotiators, making the conditions less comparable. In Study 3 there was no difference in the messages received.

### Method

#### Participants and Design

Six hundred and thirty-four participants (*Modus*_age_ = 20–24 years) from across the country completed this online study. As in Study 2, participants were randomly assigned to a (1) *high power*, (2) *low power*, (3) *low power+goal*, or (4) *low power+plan* condition. As remuneration for taking part in the study, all participants entered a lottery for a €25 gift certificate of a popular internet store.

#### Procedure

Participants were recruited on Facebook. As in Study 2, participants assumed the role of a job candidate in an online negotiation with a prospective employer about the conditions of a possible work contract. After reading the instructions, participants were asked two attention check questions and their subjectively felt power was measured for the first time (see below). Next, the self-regulation manipulation was established in the *goal* and the *plan* conditions (see below). Participants then saw a “Waiting for partner” message while the program allegedly connected participants to their opponent. Negotiations lasted for a total of three rounds of offers and counteroffers. After that, participants were told that the study’s goal was to examine early phases of negotiations and that it had not been necessary to reach a final agreement. Then, participants were asked for their absolute acceptance limit (i.e., the lowest offer from the employer they would have been willing to accept). Finally, subjectively felt power was measured a second time and participants completed a post-experimental questionnaire before being debriefed and thanked.

#### Negotiation Task

The negotiation task was similar to Study 2 with the following exceptions: participants could not send messages, the negotiation ended after three rounds (instead of six), participants were not told that the points they earned would be converted into lottery tickets, and participants were not told that only dyads with an agreement would participate in the lottery.

#### Experimental Manipulations

In contrast to Study 2 and to keep the study brief, we used only one power manipulation: High-power negotiators were told that they had numerous job offers, which they could fall back to if this negotiation ended without an agreement (Pinkley et al., 1994). In contrast, low-power parties were told that the market was very competitive and that they had no alternatives to this particular job opportunity.

Self-regulation was manipulated as in Study 2. We shortened the if-then plan to: “If my opponent makes a request, then I will not be swayed and budge from my offer in small steps only.”

#### Dependent Variables

Participants’ absolute limits served as the main dependent variable. The nature of the paradigm does not allow measuring final agreements, since negotiations were interrupted after three rounds (e.g., De Dreu and Van Lange, 1995; Sinaceur and Tiedens, 2006; Van Kleef and Côté, 2007). Absolute limits were collected as a proxy of final agreements.

In follow-up analyses, initial concessions (first counteroffers) and subsequent concessions during the negotiation process served as competing mediators. Subsequent concessions were calculated as final counteroffers minus counteroffer in round 2 (with initial concessions partialled out to remove overlap between the two mediators, see Study 2).

After instructions, we asked two attention check questions to confirm that each participant had understood the task and her/his role [e.g., “How are you supposed to imagine your situation?: (a) My chances on the labor market are good, (b) My chances on the labor market are comparable to those of my peers, (c) My chances on the labor market are poor”]. In addition, subjectively felt power was assessed with one item before and after the negotiation (“I feel/felt powerful in this role as potential employee”; 1 = *strongly disagree* to 7 = *strongly agree*). Finally, several items asked for demographic data [e.g., sex, age (assessed in categories spanning 5 years each)] and participants could report any difficulties encountered during the study in an open text box.

### Results

#### Manipulation Check

As expected, after the power manipulation, but before the negotiation, participants in the *high-power* condition felt significantly more powerful than participants in the three *low-power* conditions, *t*(348.56) = 24.46, *p* < 0.001, *d* = 2.62. The one-way ANOVA was highly significant, *F*(3,623) = 141.78, *p* < 0.001, $\eta_{p}^{2}$ = 0.41. Power ratings in the *low power+goal* and the *low power+plan* conditions did not differ from the *low-power* condition (*t*s < 1.20, *p*s > 0.250).

After the negotiation, a one-way ANOVA again revealed a significant, but less pronounced difference between the conditions, *F*(3,623) = 27.84, *p* < 0.001, $\eta_{p}^{2}$ = 0.12. Again, participants in the *high-power* condition felt significantly more powerful than participants in the three *low-power* conditions *t*(220.86) = 8.19, *p* < 0.001, *d* = 1.10. This time, however, participants in the *low power+plan* condition felt more powerful than participants in the *low-power* condition *t*(322.62) = 2.28, *p* = 0.023, *d* = 0.25, indicating that their perceptions of power increased during the process of the negotiation (see Study 1). Participants in the *low power+goal* condition did not differ significantly from participants in the *low-power control* condition, *t*(320.18) = 1.26, *p* = 0.210, *d* = 0.14.

#### Final Offers

**FIGURE 4:**
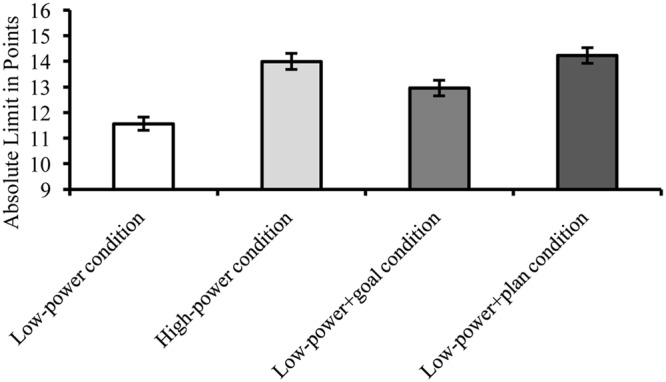
**Study 3: Absolute Limits as a function of power and self-regulatory technique (higher values indicate higher limits).** Low-power negotiators had lower absolute limits (implying lower final outcomes) than high-power negotiators and low-power negotiators with self-regulatory help. Error bars indicate ±1 *SEM*.

As expected, participants in the *low-power* condition (*M* = 11.56, *SD* = 3.45) reported lower absolute limits than participants in the *high-power* condition (*M* = 13.99, *SD* = 3.83), *t*(623) = 5.89, *p* < 0.001, *d* = 0.47. Participants in the *low power+goal* condition (*M* = 12.95, *SD* = 3.66) counteracted this power detriment. They reported higher final offers than participants in the *low-power* condition, *t*(623) = 3.40, *p* = 0.001, *d* = 0.27. However, those limits were still lower than those of high-power participants, *t*(623) = 2.45, *p* = 0.014, *d* = 0.20. Participants in the *low power+plan* condition (*M* = 14.23, *SD* = 3.79) also reported higher absolute limits than participants in the *low-power* condition, *t*(623) = 6.54, *p* < 0.001, *d* = 0.52, and did not differ from the *high-power* condition, *t*(623) = -0.57, *p* = 0.572, *d* = 0.05. Moreover, they outperformed participants in the *low power+goal* condition, *t*(623) = 3.05, *p* = 0.002, *d* = 0.24. The one-way ANOVA was highly significant, *F*(3,623) = 17.80, *p* < 0.001, $\eta_{p}^{2}$ = 0.08 (**Figure 4**). There was the expected linear trend of more ambitious absolute limits from the *low-power* condition, to the *goal* and *plan* conditions, with the latter conceding at a similar level as the *high*-*power* condition, *F*(1,624) = 52.64, *p* < 0.001 (**Figure 4**).

#### Process Analyses

**FIGURE 5:**
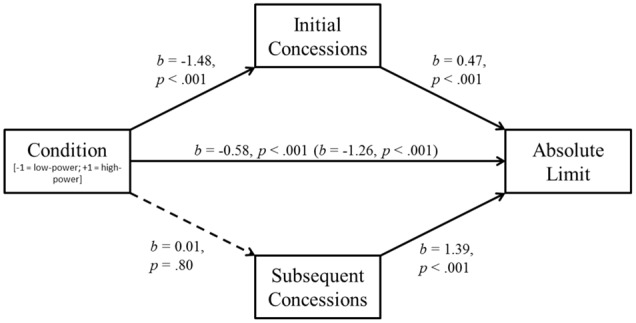
**Study 3: Mediation of the power effect by initial concessions and subsequent concessions.** The indirect path through initial concession is significant, the path through subsequent concessions is not. Total effect of power before inclusion of mediators in parentheses.

We conducted the same process analyses as in Experiment 2. There was a significant indirect effect of power on absolute limits through initial concessions, *b* = -0.69, *SE* = 0.13, BC CI_95%_ [-0.954, -0.464]. The indirect path through subsequent concessions was not significant (*b* = 0.02, *SE* = 0.08, BC CI_95%_ [-0.142, +0.169]). This finding suggests that power unfolded its effect mainly at the beginning of the negotiation (**Figure 5**).

**FIGURE 6:**
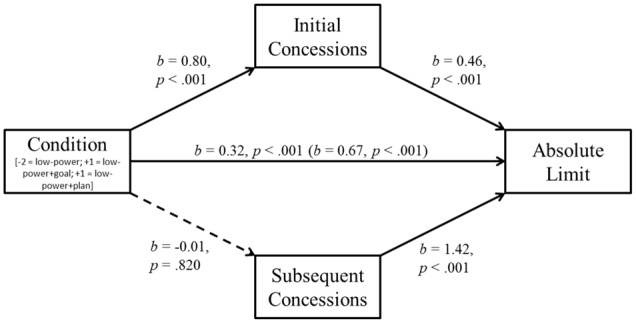
**Study 3: Mediation of the self-regulation effect by initial concessions and subsequent concessions.** The indirect path through initial concession is significant; the path through process concessions is not. Total effect of self-regulation before inclusion of mediators in parentheses.

In a second step, we again analyzed if the beneficial effect of self-regulation was mediated through initial or subsequent concessions (see Study 2). Replicating results from Study 2, there was a significant indirect effect through initial concessions, *b* = 0.37, *SE* = 0.07 BC CI_95%_ [+0.232, +0.504], whereas the indirect path through subsequent concessions was not significant, *b* = -0.01, *SE* = 0.05, BC CI_95%_ [-0.113, +0.085] (**Figure 6**).

### Discussion

Study 3 replicated the main findings from Study 2 with a larger sample. Again, participants with self-regulatory help of setting goals or forming if-then plans attenuated the low-power disadvantage. This time, low-power negotiators with additional if-then plans made even less conciliatory offers than those who were only equipped with goals. Most likely, finding this difference between the goal and the if-then conditions – an *a priori* predicted effect – was due to the much larger sample size of Study 3 (compared to Study 2). Moreover, Study 3 demonstrates that power unfolds its effect mainly at the beginning of a negotiation.

## General Discussion

Being in a low-power position represents a serious negotiation disadvantage. In Study 1, low-power parties without self-regulatory help were exploited by their more powerful opponents in a face-to-face negotiation. Study 2 revealed a similar pattern in a computer-mediated negotiation. Low-power negotiators without self-regulatory help made more conciliatory final offers to a simulated opponent than high-power negotiators. Study 3 replicated these findings in a large online sample. Given that crafting profitable deals is a key goal in negotiations, poorer negotiation outcomes by low-power parties constitute impaired goal attainment. The present findings suggest that the self-regulation techniques of setting goals and forming if-then plans can help to overcome this low power disadvantage. In Study 1, low-power negotiators who had set themselves a goal, achieved similarly high outcomes as their high-power opponents. In Study 2, both low-power parties equipped with a goal and those who additionally formed an if-then plan overcame this disadvantage. Equally, in Study 3 goals and if-then plans helped low-power participants to resist large and premature concessions to a high-power opponent. Participants who had formed an if-then plan performed better than those who had only set a goal. In sum, leading low-power negotiators to use techniques that are supposed to strengthen goal pursuit improved their goal attainment at the negotiation table (i.e., they reached more profitable negotiation outcomes).

It is important to note that self-regulatory help was pitted against multiple, established power manipulations. In Study 1, high-power negotiators were motivated by monetary incentives, benefited from a superior role, superior alternatives, and a power priming procedure. Nevertheless, negotiators overcame the low-power disadvantage if they were provided with self-regulatory help. Similarly, in Study 2 we used two power manipulations and again outcomes were linked to real world incentives. Again, self-regulatory assistance overcame the low-power disadvantage.

Studies 2 and 3 also sought to identify one process underlying the power disadvantage, as well as the positive effects of setting goals and forming plans. Study 3 demonstrated that low power unfolds its detriment mainly because of higher initial concessions. In contrast to subsequent concessions, these initial concessions statistically accounted for the low-power disadvantage (although not in the weaker powered Study 2). In both studies, initial concessions mediated the success of self-regulatory aid, whereas subsequent concessions did not. This suggests that the self-regulation interventions helped particularly in the early phases of the negotiations. These results are in line with a host of studies speaking to the importance of negotiations’ early stages (e.g., Galinsky and Mussweiler, 2001; Loschelder et al., 2014a, 2016).

In sum, the self-regulatory tools applied in the present studies led low-power negotiators to make self-serving counteroffers, to reject bad offers by their opponent, and to resist large and premature concessions. Although it may seem obvious that these are beneficial negotiation strategies, research suggests that people with low power generally have trouble initiating even simple goal-directed actions (Galinsky et al., 2003; Guinote, 2007; Steidle et al., 2013). Deliberately setting goals and forming if-then plans to engage in these behaviors are therefore promising and easily implemented tools for low-power parties.

We are optimistic that the present findings will generalize to real-life negotiations. At the outset of this article, we introduced a job applicant facing a low ball offer and raised the question how she could craft a profitable deal despite being in a low-power position. Based on our findings, this negotiator should pay close attention to not making large concessions early on, despite being in a low-power position and despite being confronted with an ambitious first offer from her high-power opponent. Setting goals and additionally forming if-then plans lend themselves to implement these negotiation strategies. Even though if-then plans did not always yield an additional benefit over goals for the low-power negotiators, they are recommendable as they are easy to implement, quick, and do not require a lot of mental resources. Consequently, they have been applied successfully in a host of different real-world contexts (e.g., Gollwitzer and Sheeran, 2006).

In both Studies 2 and 3 if-then plans included a negation (e.g., “[…] then I will not budge”). Their effectiveness appears to be at odds with evidence suggesting that negations in if-then plans can produce ironic effects by increasing the accessibility of the unwanted response (Adriaanse et al., 2011). There are a few possibilities why ironic effects did not occur in our studies. For example, ironic effects might be linked to specific behaviors. Adriaanse et al. (2011) investigated eating behavior. What we eat is largely a habit and habits are strong, automatic behavioral tendencies (Verplanken and Wood, 2006). Possibly, habits have a particularly pronounced potential to override desired actions. Another explanation lies in the nature of computer-mediated negotiations. It has been shown that negations first activate what they are supposed to prevent before they activate the desired opposite (Hasson and Glucksberg, 2006). This indicates that ironic effects might be particularly pronounced when fast responses are required. This is not the case in computer-mediated negotiations. After receiving an offer, participants had time to overthink their actions, potentially preventing ironic effects.

### Limitations and Future Directions

The present studies established that the self-regulatory techniques of setting goals and if-then plans are capable of improving negotiation outcomes of low-power parties. One limitation of the present studies is that they did not fully elucidate the psychological mechanisms underlying these beneficial effects of the self-regulation techniques. We based our case on the observation that low-power negotiators attain suboptimal outcomes and on prior findings demonstrating that people with low (as compared to high) power have difficulties to set goals and pursue them effectively (Wolfe and McGinn, 2005; Steidle et al., 2013). Accordingly, we used goal setting and if-then plans to help low-power negotiators implement broad negotiation strategies like rejecting bad offers. Admittedly, this is a rather unspecific approach: First, we do not know how exactly low power (compared to high power) influenced goal setting and goal pursuit in the present studies. Second, it remains unclear how exactly participants understood and processed the self-regulatory aid. This, equally, needs to be addressed—especially since, in contrast to earlier studies (Gollwitzer and Sheeran, 2006), only one of three studies (Study 3) demonstrated an incremental benefit of if-then plans compared to setting goals.

The process analyses reported in Studies 2 and 3 may provide some insight into why this was the case. These analyses revealed that – beyond initial concessions – subsequent concessions did not account for the self-regulatory effects on final outcomes. This indicates that participants either stopped following the plan or applied it more rigorously in the initial stages. Accordingly, future research could investigate two different approaches to maximize the if-then plan effect. First, if-then plans should be geared toward unfolding their effect throughout the negotiation (e.g., “Throughout the negotiation, if my opponent makes a request, then I will not be swayed and budge from my offer in small steps only”). Second, if-then plans could specifically address resistance to initial concessions (e.g., “At the beginning of the negotiation, if my opponent makes any request, then I will make sure not to be swayed and take a tough stance”). In both cases, detailed additional measures need to tap into negotiators understanding of the goals and plans.

On a related note, it might be argued that the goals assigned to participants were not challenging and specific enough, potentially curbing the benefits of these goals (Zetik and Stuhlmacher, 2002). However, assigning specific goals does not come without risks. In negotiations – where the motives and tactics of one’s counterpart are unclear – actors with general “do your best” goals do better than those with specific goals (Polzer and Neale, 1995). Nevertheless, investigating the relative advantages and downsides of more specific goals is a promising avenue for future research.

## Concluding Thoughts

Power is one of the most fundamental determinants for negotiation behavior and outcomes (Thompson et al., 2010). Frequently, low power is associated with excessive concessions and impaired negotiation outcomes. The question of how to overcome the low-power disadvantage has received surprisingly little attention, however. In the present research, we applied a self-regulatory perspective that understands the low-power disadvantage as impaired goal attainment. The self-regulation techniques of setting goals and forming plans helped low-power parties to attain markedly improved negotiation outcomes – particularly so by reducing their higher initial concessions.

## Author Contributions

AJ developed the research questions, studies’ concepts, and performed data collection and analysis under the supervision of MF. All authors contributed to the studies’ concrete designs. AJ drafted the manuscripts, MF and DL provided critical revisions. All authors approved the final versions of the manuscripts for submission.

## Conflict of Interest Statement

The authors declare that the research was conducted in the absence of any commercial or financial relationships that could be construed as a potential conflict of interest.

## Footnotes

1 www.statista.com (Statista.com, 2015a,b)
